# Genetic and Environmental Factors Associated with the Development of Hypertension in Pregnancy

**DOI:** 10.2188/jea.16.1

**Published:** 2005-12-20

**Authors:** Gen Kobashi

**Affiliations:** 1 Department of Health for Senior Citizens, Hokkaido University Graduate School of Medicine.

**Keywords:** Hypertension, Pregnancy, Genes, Environment, Risk Factors

## Abstract

Hypertension in pregnancy (HP), one of the most common causes of perinatal deaths, is a multifactorial disease with genetic and environmental factors involved in its etiology. We have carried out molecular epidemiologic research with the purpose of (1) identifying gene variants associated with HP in Japanese women, and (2) analyzing the genetic and environmental factors involved in the pathophysiology of the disease. Self-administered questionnaires were returned by the subjects between 1 and 6 months after delivery. The candidate genetic variants were identified by use of a PCR-RFLP method. T235 of *AGT*, C1166 of *AT1* and Asp298 of *NOS3* were respectively associated with HP, although no significant associations were found between the common genetic variants and HP in *ACE*, *FV*, *MTHFR*, *B3AR*, *TNF-A*, *PAI-1*, *GSTP1*, *mEH*, and *LPL*. In analyses using genetic, environmental and lifestyle factors, 5 factors before pregnancy and 4 factors during pregnancy were significantly associated with HP in univariate analysis. Further multivariate analysis revealed 3 factors before pregnancy, i.e. “prepregnancy BMI ≥ 24 kg/m^2^”, “family history of hypertension” and “TT genotype of *AGT*”, and 2 factors during pregnancy, i.e. “mentally stressful condition” and “salty dishes preferred”. Dividing the subjects into 2 subgroups according to whether they possessed “TT genotype of *AGT*” or not, we identified acquired risk factors before and during pregnancy for HP in each groups. The multivariate analysis identified “mentally stressful condition” as a potent significant risk factor during pregnancy in the former subgroup. However, there were no significant risk factors concerning and “mental stress” in the latter subgroup. Through further exploration of the risk factors associated with HP, we hope to provide useful suggestions about the development of new and effective preventive measures for a range of multifactorial diseases.

Hypertension in pregnancy (HP), one of the most common causes of perinatal deaths, is a multifactorial disease with genetic and environmental factors involved in its etiology. HP is believed to be caused by a combination of biological imbalance in the body (i.e., a combination of genetic variants that increases susceptibility to HP) and environmental factors. In other words, HP is triggered when the amount of interaction between genetic and environmental factors exceeds a certain level.

Recent advancement in molecular biology and epidemiology is expected to improve our understanding of the role of genetic factors in the complex etiology of HP. The analysis of both HP susceptibility genes and the lifestyle factors associated with HP can help identify the interaction between these two factors. There is an increasing need for the development of effective preventive measures for HP that takes into consideration the characteristics of individual patients.

We have carried out molecular epidemiologic research with the purpose of (1) identifying gene variants associated with HP in Japanese women, and (2) analyzing the genetic and environmental factors involved in the pathophysiology of the disease. This paper reports the results obtained so far from this research and provides a direction for future research on HP.

## Series of molecular epidemiologic studies

Patients with HP and normal controls were recruited from women aged 20-34 years with primiparous, single-fetus pregnancies delivered at Hokkaido University Hospital and its affiliated hospitals between 1993 and 1998. HP was diagnosed (following the criteria of the consensus report^1^ when at least one of the following criteria was satisfied: (1) increase of systolic blood pressure of 30 mmHg or higher, and/or (2) increase of diastolic blood pressure of 15 mmHg or higher, compared to the average value before 20 weeks of gestation, or (3) blood pressure 140/90 mmHg or higher after 20 weeks of gestation. Women with blood pressure 140/90 mmHg or higher and proteinuria before 20 weeks of gestation or at 4 weeks after delivery were excluded from the HP subjects, because they may have had latent hypertensive or renal diseases. Normal controls were women who did not experience hypertension, proteinuria, or edema during their pregnancies and for 1 month after delivery. Women with HELLP (hemolysis, liver dysfunction, low platelets) syndrome, renal disease, diabetes mellitus, amniotic volume abnormalities, or fetal anomalies were excluded from this study.

Self-administered questionnaires were returned by the subjects between 1 and 6 months after delivery. The semiquantitative questionnaire asked questions about (1) characteristics of mother and baby, (2) symptoms, (3) practices during pregnancy, (4) dietary habits, food preferences, and consumption, (5) exercise, sleep, stress, and physical burden, and (6) smoking and alcohol drinking before pregnancy and during pregnancy (the 20th to 25th gestational week).^2^ Parity, maternal age, family history of hypertension and prepregnancy BMI were determined from medical records. Pregnancies with prepregnancy BMI ≥24 kg/m^2^ were defined as prepregnancy high BMI. Informed consent for the study was obtained from every subject, following the guidelines for informed consent in epidemiologic studies,^3^ which was approved by the Institutional Review Board of Hokkaido University.

Genomic DNA was extracted from 1.0 mL of whole blood using DNA Extractor WB Kits (Wako Chemicals, Tokyo, Japan). The variant M235T of angiotensinogen gene (*AGT*), A1166C of angiotensin II type 1 receptor gene (*AT1*), Insertion/Deletion of angiotensin converting enzyme gene (*ACE*), Leiden mutation of Factor V gene (*FV*), C677T of Methylenetetrahydrofolate reductase gene (*MTHFR*), T64A of beta 3 adrenergic receptor gene (*B3AR*), Glu298Asp of endothelial nitric oxide synthase gene (*NOS3*), G-308A of tumor necrosis factor gene (*TNF-A*), 4G/5G of plasminogen activator inhibitor-1 gene (*PAI-1*), Ile105Val of glutathione S-transferase P1 gene (*GSTP1*), Tyr113His of Microsomal epoxide hydrolase gene (*mEH*), and S447X of Lipoprotein lipase gene (*LPL*) were identified by use of a PCR-RFLP method previously described, respectively, followed by 1.5% agarose electrophoresis or 10% polyacrylamide electrophoresis.^4^^-^^10^

Statistical analyses were performed after all variables were dichotomized. For example, in the analysis using *AGT* genotypes as a genetic factor, the number of T235 homozygous (TT) women was compared with that of M235 homozygous (MM) plus heterozygous (MT) women, and the answers of the questions were first transformed to binary or yes/no type answers by pooling them according to their categories (summarized in footnotes of Tables). Univariate analysis was carried out using the chi-squared test (df = 1). Yates’ correction for continuity was used when the observed number was less than 5. A stepwise method was applied to identify significant (p<0.05) factors in multivariate analysis of factors which were significantly associated with HP in univariate analysis. A multiple logistic model was applied to evaluate the odds ratios of the major risk factors. Furthermore, for tree-based analysis, dividing the subjects were divided into 2 subgroups according to whether they possessed “the TT genotype of *AGT*” (n=175) or not (n=109), and the univariate and the multivariate analyses described above were carried out for these 2 groups. All the statistical analyses were conducted by use of the Statistical Analysis System^®^ package (SAS Institute Inc., Cary, NC).

## Associations between hypertension in pregnancy and genetic variants

Table 1 shows the distributions of M235T variants of *AGT*, A1166C variants of *AT1*, and Glu298Asp variant of *NOS3*. The rate of homozygosity of T235 (TT) of *AGT* was significantly higher in HP patients than in controls among the subjects age 20-34 years.^4^ The frequency of women with AC+CC genotypes of *AT1* was significantly higher in HP cases than in controls (p<0.05) among primiparous subjects, whereas no significant differences were found among multiparous subjects.^5^ The frequency of GA+AA genotypes of *NOS3* was significantly higher in HP cases than that in controls (p<0.01). Multivariate analysis revealed that the following 4 were identified as independent potent risk factors for HP: “family history of hypertension (FH of HT)”, “TT genotype of *AGT*”, “GA+AA genotype of *NOS3*”, and “prepregnancy high body mass (BMI ≥24kg/m^2^)”, after adjusting for maternal age and parity. The odds ratios of the factors were 2.7, 2.3, 2.2 and 2.1, respectively, in a multiple logistic model (Table 2).^9^ The distributions of candidate genes for hypertension pregnancy are shown in Table 3. There were no significant associations between the common genetic variants and HP in *ACE*, *FV*, *MTHFR*, *B3AR*, *TNF-A*, *PAI-1*, *GSTP1*, *mEH*, and *LPL*.^6^^,^^7^^,^^8^^,^^10^^,^^11^

**Table 1.** Distribution of M235T variant of angiotensinogen gene, A1166C variant of angiotensin II type1 receptor gene, and Glu298Asp variant of endothelial nitric oxide synthase gene.

Diagnosis	Age (years)	Parity	No.				Frequency
				M235T of *AGT*			
	
				MM	MT	TT	TT
	
HP	20-34		89	1	8	80	90***
	35+		24	1	7	16	66
Controls	20-34		310	13	123	174	56
	35+		58	5	20	33	57

				A1166C of *AT1*			
	
				AA	AC	CC	AC+CC
HP	20-34	P=0	82	66	15	1	20*
		P>0	62	56	6	0	10
Controls	20-34	P=0	181	165	14	2	9
		P>0	134	116	15	3	13

				Glu298Asp of *NOS3*			
	
				GG	GA	AA	GA+AA

HP			152	117	35	0	23**
Controls			335	294	41	0	12

HP: Hypertension in pregnancy

*: p<0.05, **: p<0.01, ***: p<0.001 vs. controls by chi-squared test (df=1)

(Kobashi G, et al. 1999,^4^ Kobashi G, et al. 2004,^5^ and Kobashi G, et al. 2001^19^)

**Table 2.** Odds ratios (ORs) of selected risk factors existing before pregnancy for hypertension in pregnancy calculated by multivariate analysis.

	OR (95% CI)
Family history of hypertension	2.7 (1.6-4.4)**
TT genotype of *AGT*	2.3 (1.5-3.5)**
GA+AA genotype of *NOS3*	2.2 (1.3-3.7)*
Prepregnancy high body mass (BMI ≥24kg/m^2^)	2.1 (1.3-3.4)*
Maternal Age ≥35 years	-
Primiparity	-

BMI: Body mass index

*: p<0.05, **: p<0.01.

(Kobashi G, et al. 2001^9^)

**Table 3.** Distributions of variants of candidate genes for hypertension in pregnancy (%).

Genotypes	HP			Controls		
	
	AA	Aa	aa	AA	Aa	aa
*AGT*	1	19	80	4	40	56***
*AT1*	80	19	1	91	8	1*
*NOS3*	77	23	0	88	12	0**
*ACE*	42	43	16	41	47	12
*FV*	100	0	0	100	0	0
*PAI-1*	40	50	10	39	47	14
*TNF-α*	96	4	0	96	4	0
*MTHFR*	44	55	11	39	46	15
*LPL*	76	22	2	76	23	1
*B3AR*	70	24	6	70	28	2
*GSTP1*	72	28	0	74	26	0
*mEH*	24	34	42	26	29	45

HP: Hypertension in pregnancy

A:wild type, a: mutant type

*: p<0.05, **: p<0.01, ***: p<0.001

(Kobashi G. 2003^11^)

## Confoundings of genetic, environmental and lifestyle factors in the development of hypertension in pregnancy

Table 4 shows 6 factors before pregnancy and 4 factors during pregnancy, which were significantly (p<0.05) associated with HP in univariate analysis. Further multivariate analysis revealed 3 factors before pregnancy, i.e. “prepregnancy BMI ≥24kg/m^2^”, “family history of hypertension” and “TT genotype of *AGT*”, and 2 factors during pregnancy, i.e. “mentally stressful condition” and “salty dishes preferred” that were potent independent risk factors for HP.^11^

**Table 4.** Risk factors before and during pregnancy for hypertension in pregnancy.

Factors	Positive answers (%)		Odds ratio (95% CI)	
	
	Cases	Controls	Univariate	Multivariate
	Factors before pregnancy	
High body mass index (BMI ≥24 kg/m^2^)	29.4	9.0	4.2 (2.1-8.5)***	4.8 (1.5-6.0)***
Family history of hypertension	39.7	20.5	2.6 (1.4-4.6)**	3.0 (1.5-6.0)***
TT genotype of *AGT*	75.0	59.9	2.0 (1.1-3.7)**	2.6 (1.3-5.0)**
Poor relationship with husband’s parents^†^	29.4	15.2	2.3 (1.2-4.4)**	-
Mentally stressful condition^†^	27.9	16.6	2.0 (1.0-3.7)*	-
GA+AA genotypes of *NOS3*	23.5	15.2	1.7 (0.9-3.4)	

	Factors during pregnancy	
Mentally stressful condition^†^	54.4	31.3	2.6 (1.5-4.6)^***^	3.5 (2.1-10.6)^***^
Salty dishes preferred^†^	36.8	19.7	2.4 (1.3-4.4)^**^	2.5 (1.8-6.7)^**^
Poor relationship with husband’s parents^†^	23.5	12.3	2.2 (1.1-4.4)^*^	
Physically demanding work^†^	47.1	33.2	1.8 (1.0-3.1)^*^	

There were 71 cases and 213 controls.

Factors with p<0.1 in univariate analysis were subjected to multivariate analysis and are shown.

Odds ratios are shown only when a significant difference existed (*p<0.05, **p<0.01, ***p<0.001).

†: Five possible answers were: a, yes; b, somewhat; c, neutral; d, not much; and e, not at all. In this table, a+b was compared with c+d+e, with a+b considered a risk factor

CI: confidence interval

*: p<0.05, **: p<0.01, ***: p<0.001

(Kobashi G, et al. 2002^2^)

Dividing the subjects into 2 subgroups according to whether they possessed “TT genotype of *AGT*” (n=175) or not (n=109), we identified acquired risk factors before and during pregnancy for HP that were significant (p<0.05) by univariate and multivariate analyses. Tables 5 and 6 show significant risk factors for HP in each subgroup. The multivariate analysis identified “mentally stressful condition” as a potent significant risk factor during pregnancy in the former subgroup. However, there were no potent significant risk factors concerning and “mental stress” in the latter subgroup.^11^

**Table 5.** Risk factors during pregnancy for hypertension in pregnancy in the subgroup “TT genotype of *AGT*”.

Factors	Positive answers (%)		Odds ratio (95% CI)	
	
	Cases	Controls	Univariate	Multivariate
	Factors before pregnancy	
High body mass index (BMI ≥24kg/m^2^)	27.5	12.1	2.7 (1.2-6.2)*	3.7 (1.4-9.7)**
GA+AA genotypes of *NOS3*	31.4	16.3	2.4 (1.1-5.0)*	3.3 (1.3-8.4)*
Family history of hypertension	35.3	15.8	2.9 (1.4-6.2)**	2.9 (1.3-6.8)*
Poor relationship with husband’s parents^†^	31.4	15.3	2.5 (1.2-5.4)*	-

	Factors during pregnancy	
Mentally stressful condition^†^	54.9	30.7	2.8 (1.4-5.4)**	3.8 (1.8-8.3)***
Poor relationship with husband’s parents^†^	25.5	12.9	2.3 (1.0-5.2)*	-
Salty dishes preferred^†^	35.3	20.6	2.1 (1.0-4.4)**	-

There were 51 cases and 124 controls in the subgroup.

Factors with p<0.1 in univariate analysis subjected to multivariate analysis and are shown.

Odds ratios are shown only when a significant difference existed.

†: Five possible answers were: a, yes; b, somewhat; c, neutral; d, not much; and e, not at all. In this table, a+b was compared with c+d+e, with a+b considered a risk factor

CI: confidence interval

*: p<0.05, **: p<0.01, ***: p<0.001

(Kobashi G, et al. 2002^2^)

**Table 6.** Risk factors during pregnancy for hypertension in pregnancy in the subgroup “MM+MT genotypes of *AGT*”.

Factors	Positive answers (%)		Odds ratio (95% CI)	
	
	Cases	Controls	Univariate	Multivariate
	Factors before pregnancy	
High body mass index (BMI ≥24 kg/m^2^)	35.3	4.8	10.8 (2.6-44.3)*	10.5 (2.6-43.1)**

	Factors during pregnancy	
Salty dishes preferred^†^	41.2	15.6	3.8 (1.2-11.9)*	-

There were 20 cases and 89 controls in the subgroup.

Factors with p<0.1 in univariate analysis subjected to multivariate analysis and are shown.

Odds ratios are shown only when a significant difference existed.

†: Five possible answers were: a, yes; b, somewhat; c, neutral; d, not much; and e, not at all. In this table, a+b was compared with c+d+e, with a+b considered a risk factor

CI: confidence interval

*: p<0.05, **: p<0.01, ***: p<0.001

(Kobashi G, et al. 2002^2^)

## Candidate genes and their possible pathogeneses in the development of hypertension in pregnancy

*AGT* variant was the first gene variant found to be associated with the occurrence of HP. The amino acid located at position 235 in the AGT protein is known to be either methionine (M235) or threonine (T235). Of the two *AGT* alleles, T235 has been found to occur at a higher frequency among women with HP than among women with normal pregnancy outcome.^12^ Genetic variants of proteins from the rennin-angiotensin system, blood coagulation system, and intravascular system have been thoroughly studied in terms of their association with HP.^13^ To date, the *AGT* M235T variant, the *AT1* A1166C variant, and the *NOS3* Glu298Asp variant have been found to be associated with HP in Japanese women.^4^^,^^5^^,^^9^

Several factors are believed to be associated with HP, including pressor/depressor mechanisms, the blood coagulation system, lipid metabolism, the intravascular system, placenta, abdominal wall flexibility, physical/psychological stress, and the nervous system. Consequently, a number of gene variants have been examined in terms of their contribution to HP. However, due to genetic differences among races, a gene variant that shows association with HP in Caucasian women may show no significant association with HP in Japanese women.^7^ Even when a certain gene variant is found to be associated with HP in Japanese women, the frequency of disease occurrence may vary across patients with different baseline characteristics.^8^^,^^14^ Despite the genetic differences between the general Japanese population and the general Caucasian population, the contribution of the *AGT* variant to the onset of HP has been demonstrated and proven among Japanese as well as Caucasian populations. A later study has demonstrated that *AGT* M235T polymorphism is in tight linkage disequilibrium with a molecular variant in the proximal promoter of the *AGT*, which consists of an adenine instead of a guanine six-nucleotide upstream from the site of transcription initiation (G-6A). In other words, M235 variant is a marker for -6G and T235 variant is a marker for -6A.^15^ Furthermore, according to an *in-vitro* study, -6A was associated with a lower promoter activity than -6G.^15^ T235 (-6A) is reported to be associated with an inadequate trophoblastic invasion of the uterine spiral arteries and narrowing of spiral arterioles in early pregnancy, which can provoke the onset of HP.^16^

According to a previous study carried out among Caucasian women to examine the association between the *AGT* T235 variant and preeclampisa (PE) (hypertension with proteinuria), the *AGT* T235 allele frequency was significantly higher among primiparous women with PE with respect to the control group (65% vs. 40%).^12^ On the other hand, in the Japanese population, the T235 variant was strongly associated with the incidence of HP, regardless of parity. Furthermore, T235 allele appears at a higher frequency among the Japanese population (94% in patients with HP and 76% in the control) than among the Caucasian population.^4^ *FV* Leiden mutation is the mutation in the blood coagulation *FV*, causing activated protein C resistance. Known as the most common genetic risk factor for thrombosis, it is present in 10% of the Caucasian women with HP and 4% of the Caucasian women with normal pregnancy,^17^ but is very rare among the Japanese population.^7^ Thus, two main genetic factors may be associated with the onset of HP among the Caucasian population, namely, the *AGT* variants (PE among primiparous women) and the *FV* Leiden mutation. On the other hand, HP among the Japanese population is predominantly associated with the *AGT* variants.

HP has long been known to have various forms and causes. Many researchers currently believe that the symptoms of HP are the products of the interaction of various genetic as well as environmental factors. In fact, what is clinically termed HP is actually a multifactorial syndrome comprising a heterogeneous group of diseases. Thus, results obtained from a study on genetic and environmental risk factors for HP may provide not only a useful insight into the etiology and pathology of this multifactorial disease but also a basis for classifying HP. It is neither practical nor appropriate to use pedigree analysis or sib-pair analysis to identify candidate genes involved in the development of HP, because the population at risk is limited to pregnant women. The use of a case-control study is more appropriate in this respect. Furthermore, it is important to remember that even when a certain gene is found to have no statistically significant association with the development of HP among pregnant women in general, a significant association may be found when the association is assessed in patients with specific groups. Furthermore, caution is needed when interpreting the results obtained from a multivariate analysis because a multivariate approach can only detect potent risk factors for general subjects. For example, no association was initially found between the IV+VV genotypes of *GSTP1* and HP among pregnant women in general. However, this genotype turned out to be significantly associated with the development of HP among primiparous women with severe HP who belonged to one of the following three groups: (1) a group of women in older age, (2) GA+AA genotypes of *NOS3* (this genotype is known to be associated with HP), and (3) MM+MT genotypes of *AGT* (this genotype is known to be associated with reduced risk of toxipathy)^10^ (data are not shown in the present paper). *GSTP1* belongs to a family of drug metabolizing enzymes. Polymorphism of the *GSTP1* results in an amino acid substitution (isoleucine to valine) at codon 105, which causes a substrate-specific functional change in the activity of the enzyme. When this genotype interacts with the *NOS3* variant and/or with older age, the NO generation may decrease, which potentially triggers HP. On the other hand, the *AGT* TT genotype has been reported to be associated with vasculopathy of the spiral arteries of the placental bed^16^, and is possibly an independent risk factor for HP.^10^

## A possibility of tailor-made/individualized medicine for hypertension in pregnancy

**Figure 1.**
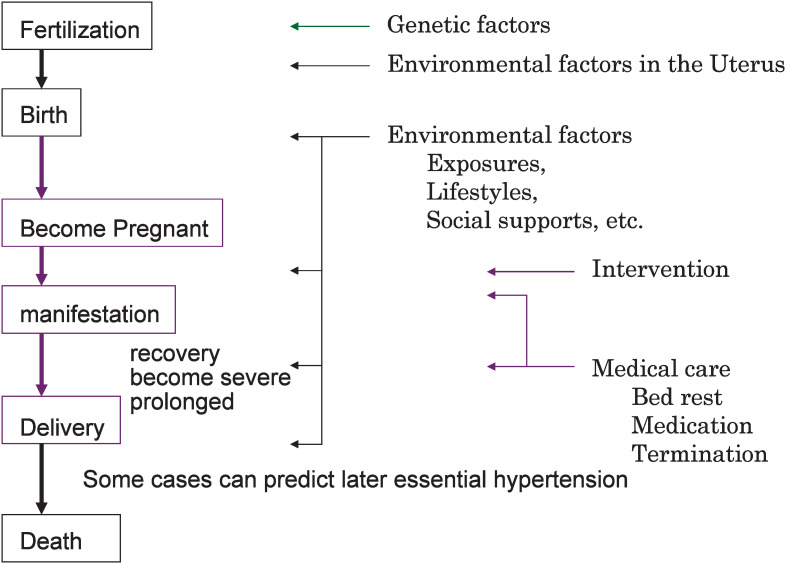
Natural history of hypertension in pregnancy

Thus, it can be concluded that several factors, such as genetic, environmental, and age factors, are involved in the pathogenesis of HP (Figure 1). Preventive measures for HP should address environmental as well as lifestyle-related risk factors during pregnancy and/or the pre-pregnancy period. In fact, it is widely acknowledged that consulting an obstetrician for advice regarding appropriate lifestyle during pregnancy is effective to some extent in reducing the risk of HP. However, due to interindividual genetic differences, a lifestyle-related factor that is identified as a strong risk factor for HP among a certain group of individuals may have no significant association with HP risk among other groups of individuals. The advancement of molecular biology may potentially improve our understanding of diseases/conditions caused by the interaction of multiple genes or a combination of genetic and environmental risk factors and it may also contribute to the development of disease prevention and health promotion measures tailored to a specific genotype.^2^

**Figure 2.**
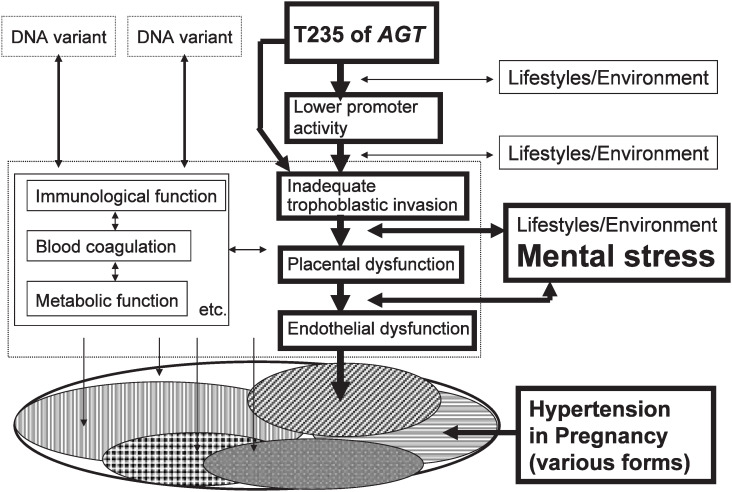
Contribution of T235 of *AGT* in the development of Hypertension in Pregnancy

The present study shows that lifestyle-related risk factors for HP vary greatly by the *AGT* genotypes, a fact which reflects the multifactorial nature of the disease. The *AGT* TT genotype is reported to be associated with an inadequate trophoblastic invasion of the uterine spiral arteries and a narrowing of spiral arterioles in early pregnancy. Coupled with the effect of physical and psychological stress during pregnancy, such factors could lead to decreased blood flow in the placenta, potentially triggering HP (Figure 2). In view of these facts, the *AGT* genotyping may be useful for developing individualized measures for relieving physical/psychological stress during pregnancy to prevent HP among those women whose risk for HP is seemingly low as measured by conventional criteria.

The goal of tailor-made/individualized medicine is to use genotyping technology to provide effective preventive measures against a range of multifactorial diseases, including cancer, strokes, myocardial infarction, and diabetes. An analytical model constructed in the present study to examine the contribution of genetic and environmental factors to HP can be applied to other multifactorial diseases as well, which could be useful for understanding the pathogenesis of the disease in question and for developing the appropriate preventive measures. However, there are several hurdles to overcome before genotyping technology can be applied for HP prevention and control, which include, (1) developing a proper genotyping technology, (2) establishing appropriate epidemiologic methods, (3) developing ethics guidelines, (4) establishing appropriate laws and regulations, (5) providing education on advantages and disadvantages of genotyping, and (6) promoting the provision of genetic counseling. However, HP research has the following advantages:

(1) Because almost all pregnant women in Japan visit a doctor for regular prenatal care during pregnancy, information regarding pregnant women’s lifestyle can be obtained with relative ease and patients have the opportunity to obtain feedback and advice on appropriate lifestyle through prenatal care visits;
(2) Because it takes less than 10 months to obtain certain pregnancy outcomes, the study results can be obtained in a short period of time; and
(3) The involvement of obstetricians may facilitate the informed consent process.

Through further exploration of the risk factors associated with HP, we hope to provide useful suggestions about the development of new and effective preventive measures for a range of multifactorial diseases.
